# Alcohol Consumption in Rheumatoid Arthritis: A Path through the Immune System

**DOI:** 10.3390/nu13041324

**Published:** 2021-04-16

**Authors:** Vugar Azizov, Mario M. Zaiss

**Affiliations:** 1 Department of Internal Medicine 3—Rheumatology and Immunology, Friedrich-Alexander Universität Erlangen-Nürnberg (FAU) and Universitätsklinikum Erlangen, 91054 Erlangen, Germany; vugar.azizov@uk-erlangen.de; 2 Deutsches Zentrum für Immuntherapie, Friedrich-Alexander Universität Erlangen-Nürnberg (FAU) and Universitätsklinikum Erlangen, 91054 Erlangen, Germany

**Keywords:** alcohol, acetate, acetaldehyde, rheumatoid arthritis, RA

## Abstract

Benefits and harms of different components of human diet have been known for hundreds of years. Alcohol is one the highest consumed, abused, and addictive substances worldwide. Consequences of alcohol abuse are increased risks for diseases of the cardiovascular system, liver, and nervous system, as well as reduced immune system function. Paradoxically, alcohol has also been a consistent protective factor against the development of autoimmune diseases such as type 1 diabetes, multiple sclerosis, systemic lupus erythematosus, and rheumatoid arthritis (RA). Here, we focused on summarizing current findings on the effects of alcohol, as well as of its metabolites, acetaldehyde and acetate, on the immune system and RA. Heavy or moderate alcohol consumption can affect intestinal barrier integrity, as well as the microbiome, possibly contributing to RA. Additionally, systemic increase in acetate negatively affects humoral immune response, diminishing T_FH_ cell as well as professional antigen-presenting cell (APC) function. Hence, alcohol consumption has profound effects on the efficacy of vaccinations, but also elicits protection against autoimmune diseases. The mechanism of alcohol’s negative effects on the immune system is multivariate. Future studies addressing alcohol and its metabolite acetate’s effect on individual components of the immune system remains crucial for our understanding and development of novel therapeutic pathways.

## 1. Introduction

Alcohol is one of the most widely consumed substances worldwide [1,2]. Due to its anxiolytic and addictive properties, alcohol is also one of the most abused substances. Alcohol is readily absorbed and metabolized in the body. It is first metabolized to acetaldehyde by alcohol dehydrogenase (ADH), then acetaldehyde is metabolized to acetate by aldehyde dehydrogenase (ALDH) by various cells of the body. Acetaldehyde is particularly toxic, but its lifetime is limited as it is readily metabolized further to acetate [3,4]. Fast metabolism of alcohol leads to increased serum acetate levels in chronic alcohol drinkers [5,6]. Due to alcohol’s fast metabolism to toxic acetaldehyde, then important metabolic intermediary such as acetate, the interest of its effects on human health has risen. Diseases such as high blood pressure; heart disease; liver disease; cancers of the mouth, throat, liver, etc.; and mental health issues, including addiction, are examples of alcohol-mediated disease [2,7,8]. Another of alcohol’s important unintended effects is on the immune system. Any level of alcohol consumption, whether acute or chronic, has immunomodulatory effects. Both arms of the immune system, innate and adaptive, are affected by alcohol consumption [9,10]. With this in mind, many studies have been undertaken to address a possible link between autoimmune diseases such as RA and alcohol use. Criteria for classification of moderate and heavy alcohol consumption generally stayed within the guidelines of National Institute on Alcohol Abuse and Alcoholism (NIAAA) and were based on dietary guidelines for Americans (shown in Table 1) or were more restricting [11].

RA is a systemic autoimmune disease primarily affecting joints and causing a progressively worsening pain, swelling, deformation, and articulation. The effects of RA extend to pulmonary, cardiovascular, skeletal, and nervous systems [12]. RA affects about 0.69 per cent of the global population [13]. Hence, it is one of the major causes of disability, leading to reduction in life expectance, secondary health complications, and socioeconomic damage [14]. While the exact mechanism of the initiation of the disease is not known, both environmental and genetic factors have been implicated in RA [12,15,16]. As RA is multifactorial and complex, both innate and adaptive immunity drive the progression of the disease. This is evidenced by the existence of anti-citrullinated protein antibody (ACPA), as well as antibodies against IgG Fc called rheumatoid factor (RF), in the serum of RA patients [17,18]. In parallel to adaptive immunity, innate immune cells such as neutrophils, macrophages, NK cells, and mast cells have been found in synovial fluids of RA patients [12]. Macrophages, as resident professional APCs, have a central role in RA progression by antigen presentation, inflammatory cytokine (e.g., IL-1, -6, -12, -15, -18, -23, tumor necrosis factor (TNF)-α) and destructive molecule production (e.g., prostanoids, reactive oxygen intermediates, nitrogen intermediates) [12]. Repertoire of molecules produced by macrophages suggests that these are inflammatory or M1 macrophages [19]. Additionally, cytokines produced by macrophages (e.g., IL-1, IL-6) also activate osteoclasts and cause bone resorption in the joints. As a result, joints are severely affected limiting articulation of limbs, pain, and swelling. Multiple avenues of treatment have been undertaken from blocking TNF-α, IL-1R, IL-6R, CD20, CD80, and CD86 [12].

Multiple studies have correlated alcohol consumption with reduced disease activity in RA. Analysis of two Scandinavian case–control studies revealed a significant dose-dependent reduction in risk of RA in alcohol consumers [20]. Another study confirmed alcohol’s protection against RA in a dose-dependent manner, but in addition identified this phenomenon to be more protective in the ACPA^+^ group than in ACPA^−^ according to serum CRP levels, DAS28 score, pain visual analogue scale, modified HAQ, and modified Larsen score [21]. It was also elucidated that alcohol’s protective effects were significant in female RA patients and not in males [22]. A metanalysis of all studies up to the year 2013 solidified alcohol’s protection against RA, with this protection being significant only in the ACPA^+^ patients rather than ACPA^−^ [23]. In the following years, further studies linked the benefits of alcohol consumption to the ACPA^+^ female population and also observed alcohol’s dose-dependent effects [24,25]. In 2019, Hedström and colleagues brought a new perspective to studies evaluating correlation between alcohol use and protection against RA. They argued that studies of such diseases should take into account environmental and genetic factors. As such, they discovered alcohol’s protective effects both in ACPA^+^ and ACPA^−^ RA risk, taking into consideration alcohol use, smoking, and presence of human leucocyte antigen (HLA)-DRB1-shared epitope (SE) [26]. Interestingly, in older adults with light alcohol consumption, characterized as an intake of one to seven drinks per week, there was an observed reduction in IL-6 and C-reactive protein (CRP) levels [27]. In retrospect, as early as 1996, ethanol treatment of human monocytes caused a reduction in TNF-α and IL-1β at mRNA and protein levels, while causing an increase in IL-10 and TGF-β [28]. Later, reduction in proinflammatory cytokines, as well as NF-κB expression in human monocytes, was shown to be only upon acute alcohol consumption or exposure to equivalent amounts in vitro [29]. Potent effects of alcohol on autoimmunity have also been observed in other diseases such as systemic lupus erythematosus (SLE), autoimmune diabetes, Graves’ hyperthyroidism, and autoimmune hypothyroidism [30,31,32,33,34]. Of note, nutrition in general has been shown to have strong effects on autoimmunity; in a study of alcohol-use and SLE, there was a significant correlation of wine but not beer consumption as a protective factor [31,35]. A recent review of the possible links between different beverages and RA discussed one of the compounds found in wine, resveratrol, inhibiting downstream mechanisms of TNF receptor [36].

On a quest to identify the specific mechanism by which alcohol acts upon immune system, one must take into consideration not only alcohol, but also the products of alcohol’s metabolism. For example, acetaldehyde, the first metabolite of alcohol, in alcoholic liver disease (ALD) patients has been shown to negatively affect tight junction molecules in intestinal epithelium, and consequently cause increased serum LPS, which is correlated with TNF receptor levels as well as increased disease activity [37,38,39]. Recently, in our laboratory, we were able to show that modulation of intestinal tight junction can affect onset of RA in the preclinical collagen-induced arthritis (CIA) model of RA [40]. Another metabolite of alcohol, acetate, is a central molecule in cellular metabolism, post-translational modifications, and transcription in its biologically active acetyl-CoA form [41]. Alcohol has been shown to contribute to increased acetylation of histones in the brain upon alcohol consumption [42,43]. In addition, a direct treatment of human neuronal cell lines with alcohol exhibited increased reactive oxygen species (ROS) and increased expression of HDAC2 [44]. On the other hand, a direct supplementation of mouse feed with acetate inhibited HDAC2 expression and activity while also increasing histone acetylation [45]. It is possible that in vitro direct treatment of cell lines with alcohol does not match accumulation of alcohol’s metabolites as efficiently as in vivo supplementation. As we have now evidenced alcohol’s ability to assert its effects via acetate, it is also important to remember that upon alcohol consumption blood acetate levels are also increased. In addition, it has been shown that acetate can modulate the immune system and, specifically, T cells [46,47,48]. As alcohol consumption is possibly one of largest contributors to blood acetate levels, bacterial fermentation of dietary fiber by the intestinal microbiota also serves as a source of short-chain fatty acids including acetate [49]. For example, in scope of multiple sclerosis (MS), intake of pro- or pre-biotics was found to positively affect disease severity [50]. Low-dose alcohol feeding of mice in a preclinical model for MS altered the microbiota in sex-dependent manner protecting against experimental autoimmune encephalomyelitis (EAE) [51]. In the RA preclinical CIA model, both alcohol and acetate consumption has been shown to ameliorate disease severity [52,53,54]. In 2007, Jonsson and colleagues observed a significant reduction in synovitis and erosion, reduction in TNF-α and MIP1-α produced by spleenocytes, and increased bone mineral density, ultimately leading to a decrease in incidence and severity of arthritis in alcohol-fed CIA mice [52]. Of course, we must delineate the effects of each and every metabolite of alcohol and of alcohol itself in order to better understand the complex network of affected parts of the immune system ultimately contributing to observed protective effects of consumed alcohol. Here, we review known effects of alcohol and alcohol’s metabolites, acetaldehyde and acetate, on the immune system within the scope of autoimmunity, namely, RA.

## 2. The Effect of Alcohol and Alcohol Metabolites on the Immune System

### 2.1. The Effect of Alcohol on the Immune System

Although measuring direct effects of alcohol in vivo can be challenging, one of the ways it is delineated from the effects of other metabolites of alcohol is by decreasing the time between alcohol administration and quantification of parameters. Upon acute alcohol intoxication of people, peripheral blood lymphocytes were shown to upregulate MHC-I molecules [55]. In the antigen presentation front, another group was able to show that spleenocytes of ethanol-fed C57BL/6 mice had diminished antigen presentation capacity in native hen egg lysozyme (HEL), hapten-conjugated HEL, and a short fragment of HEL (amino acids 51–60) immunizations [56]. Dendritic cells (DC) are one of the professional APCs that also play a key role in self-tolerance and have been found in the synovium of RA patients, contributing to proinflammatory process [12]. Mandrekar and colleagues were able to show that alcohol-exposed DCs produced less IL-12, expressed reduced levels of costimulatory molecules CD80 and CD86, and primed CD4^+^ T cells that were hyporesponsive to secondary untreated DC stimulation [57]. Following this finding, alcohol-fed mice were shown to have dose-dependent decrease in plasmacytoid DCs (pDCs) and myeloid DCs (mDCs), along with decreased CD40 expression and IL-12 production [58]. Later, Fan and colleagues showed that chronic alcohol feeding did not affect antigen uptake or processing, but these DCs exhibited decreased T cell activation capacity due to decreased costimulatory molecules, CD80, CD86, CD40, and cytokine production [59]. Langerhans cells are also affected by alcohol consumption as alcohol-fed mice exhibited decreased cell density and migration to lymph nodes (LN), but interestingly showed no change in MHC-II or CCR7 expression [60].

Macrophages and monocytes are amongst the cells of the immune system affected by alcohol consumption that contribute to RA disease progression by secreting pro-inflammatory cytokines such as TNF-α, IL-6, GM-CSF, IL-15, IFN-α/β, VEGF, FGF, CC, and CXC chemokines, ultimately fueling synovial destruction [12]. Nitric oxide (NO) is another compound produced by macrophages, neutrophils, and natural killer (NK) cells, and adds to RA progression [12]. NO has been shown to inhibit aldehyde dehydrogenase (ALDH) as well as react directly with ethanol to form ethyl nitrite [61]. Acute ethanol exposure has also been shown to reduce Kupffer cell NO production [61]. Upon alcohol intake in vivo, human monocytes exhibit activation of STAT1/3, leading to STAT-dependent induction of suppressors of cytokine signaling (SOCS) and downregulation of IL-6, IFN-α, and IFN-γ [62]. Furthermore, ex vivo acute ethanol treatment of human peripheral monocytes resulted in decreased p65 phosphorylation and consequently inhibited NF-κB DNA binding [63]. Interestingly, chronic ethanol exposure of human peripheral blood mononuclear cells (PBMC) and mouse macrophage cell lines (J774) induced reactive oxygen species and NLRP3 inflammasome hyperactivation [64]. The authors also came to a conclusion that the metabolites of alcohol, namely, acetaldehyde is the cause of NLRP3 hyperactivation and increased IL-1β secretion [64]. Similar findings on alcohol’s effect on NLRP3 hyperactivation was found in human myeloid leukemia cells (U937) induced with monosodium urate (MSU) crystals [65]. Contrary to earlier findings, in a recent finding, human PBMC-derived macrophages treated with ethanol and stimulated with Paracoccidioides brasiliensis yeast cells exhibited reduced capacity to activate T cells, favoring Th2 differentiation over Th1 and Th17 cells and reducing HLA-AB, HLA-DR, CD80, CD86, IL1β, IL-6, and ROS production [66]. Interestingly, in the same study, sodium acetate treatments in parallel to ethanol treatment exhibited the same efficacy as the highest concentration of ethanol (150 mM) [66].

In line with innate immunity, NK cells are increased in the synovial fluids of RA patients, contributing to disease by pro-inflammatory cytokine secretion and bone destruction [67]. Alcohol also affects NK cells; one study concluded that the deficiencies in NK cell function observed in chronic alcohol drinkers could be due to cell loss with a reduction in CD56^+^ CD45RA^+^ NK cells by >60% rather than direct effect of alcohol on NK cells [68]. However, this interpretation should be taken carefully, as the study was performed in the scope of alcoholic liver disease during an episode of alcoholic hepatitis. Meanwhile, invariant natural killer T (iNKT) cells in alcohol-consuming mice demonstrated increased cell maturation and higher IL-12 and IFN-γ production, ultimately favoring Th1 immune response [69]. The mucosa-associated invariant T (MAIT) cell population of the gut have also been shown to be reduced, along with decreased expression of IFN-γ and TNF-α upon alcohol-induced dysbiosis [70]. Alcohol also affects granulopoiesis by preventing stem cell antigen 1 (Sca-1) directed proliferation during bacteremia [71].

Alcohol modulates components of adaptive immunity: T cells, B cells, plasma cells, antibody production, and B cell development in the bone marrow [72]. Alcohol has been shown to affect the thymocyte maturation by causing a decrease in CD8^+^ CD4^−^ T cells in the thymus and lamina propria of chronic alcohol-consuming mice [73]. In humans, peripheral lymphocytes produced decreased levels of IFN-γ in steady and mitogen-induced states upon in vitro treatment with ethanol [74]. In alcohol-fed steady state mice, there was a marked decrease in IFN-γ-producing T cells, decreased numbers of B and NK cells, and reduced MHC-II expression by B cells, as well as increased IgE levels [75]. Interestingly, human peripheral mononuclear cells obtained from chronic alcohol drinkers did not exhibit a difference in Th2/Th1 ratio but also showed increased IgE levels [76]. Alcohol-fed mice infected with Klebsiella pneumoniae showed decreased survival, along with decreased IL-12 and IFN-γ, but also increased IL-10 and TNF-α in whole-lung homogenates [77]. In rats, acute alcohol intoxication inhibited LPS-induced TNF in bronchoalveolar lavage, but this inhibition faded with chronic alcohol feeding [78]. In alcohol-fed aldehyde dehydrogenase 2 (ALDH2) knockout mice, alcohol was shown to inhibit glucose metabolism possibly through increased corticosterone levels [79]. In the same article, the authors also showed that acetaldehyde exposure inhibited human peripheral IFN-γ production when stimulated by phytohemagglutinin [79]. During a viral infection by murine influenza virus, chronic alcohol-consuming mice exhibited decreased survival; increased pulmonary viral load; decreased total and virus-specific CD8^+^ T cell numbers, but not percentages; and a marked decrease in IFN-γ-producing CD8^+^ T cells at 8 weeks post-infection [80].

In summary, both innate and adaptive immune cells have been reported to be affected upon alcohol exposure. Decreased MHC-I, MHC-II, proinflammatory cytokines, co-stimulatory molecule expression, localization, and migration cause an overall suppression of immune system surveillance, antigen-presentation, and T cell activation.

### 2.2. The Effects of Acetaldehyde on the Immune System

The first metabolite of alcohol, acetaldehyde, generated by alcohol dehydrogenase (ADH), can react with lysine groups forming Schiff-base adducts, therefore inhibiting lysine-dependent enzymes [81]. It was found that chronic alcohol drinkers harbored acetaldehyde-modified antigens on erythrocytes and in bone marrow aspirates [82]. Furthermore, acetaldehyde-treated hepatic cell line exhibited reduced MHC-I presentation of HBV virus, shown to be due to suppression of antigen processing and formation of peptide loading complex in response to IFN [83]. Interestingly, such adduct formations have also been detected in RA. As such, in a clinical study, Mikuls and colleagues demonstrated that RA patients harbor higher concentrations of anti-malonylaldehyde-acetaldehyde (MAA) antibodies in joints [84]. In the same year, another group was able to show that RA patients exhibit increased anti-malondialdehyde modification (MDA) IgG levels correlating with serum TNF-α, IL-6, and CRP [85]. As recent as 2020, Mikuls and colleagues discovered that anti-MAA antibodies are elevated prior to RA diagnosis but seemingly after ACPA and RF, possibly solidifying the role of anti-MAA antibodies in transition from preclinical to clinical severity [86]. At the molecular level, acetaldehyde treatment of intestinal Caco-2 cell line caused decreased protein tyrosine phosphatase (PTP) 1B, PTP1C, and PTP1D activity, driving increased ZO-1, E-cadherin, and β-catenin phosphorylation affecting tight junction integrity [87,88].

In summary, acetaldehyde modifications of self-proteins are able to induce autoimmunity and dysregulate the tight junction in intestinal epithelium, contributing to increased inflammation. Moreover, although MHC-I antigen presentation is negatively affected, future studies on acetaldehyde’s effect on MHC-II antigen presentation would be necessary. Meanwhile, the fact that antibodies against modified antigens are detected after ACPA and RF could mean these antibodies contribute to RA progression later but before diagnosis.

### 2.3. The Effects of Acetate on the Immune System

Acetate and its functionally active version, acetyl-CoA, are potent effectors of cellular metabolism, transcriptional profile, and proteome [89]. In neuroblastoma, hypoxia induced DNA hypermethylation and hence reduced DNA accessibility, which was reversed upon acetate treatment [90]. In glioblastoma cells, increased ATP citrate lyase (ACLY) dependent acetyl-CoA levels caused increased histone 3 acetylation specifically at the genes promoting adhesion and migration [91]. There are limited specific studies of acetate’s effect on immune cells. Oral tolerance induction and protection against food allergies were void in GPR-43 knockout mice due to inadequate mucosal germinal center and T_FH_ response, as well as recruitment of CD103^+^ DCs [92]. Neutrophil extracellular trap (NET) formation by PMA-induced neutrophils exposed to elevated acetate levels (e.g., >0.5 mM) have been shown to be inhibited [93]. Activating CD4^+^ T cells are highly dependent upon aerobic glycolysis for increased pool of intracellular acetyl-CoA, which increases histone acetylation and hence leads to higher IFN-γ production [94]. CD8^+^ T cells that infiltrate tumors are subject to a tumor microenvironment that has decreased oxygenation and restricted glucose levels [95]. It was shown that effector CD8^+^ T cells are highly dependent upon aerobic glycolysis and must compete against tumor cells [96]. CD8^+^ T cells in a glucose-restricted environment produce less IFN-γ [97]. In such nutritionally scarce environments, elevated acetate levels have been shown to rescue IFN-γ production by CD8^+^ T cells [98]. In mice, acetate levels were found to significantly increase upon pathogenic bacterial infection, both systemically and locally. At the sites of infection, acetate reached concentrations of 5 mM as early as 4 h post-infection. Memory CD8^+^ T cells were found to produce increased levels of IFN-γ at the early stages of immune response [99]. At later stages of immune response, memory CD8^+^ T cells reduced ACSS enzymatic activity upon TCR stimulation and reduced Ca^2+^ influx, hence blocking pro-inflammatory response and avoiding immune response pathologies [100].

Altogether, exposure to elevated acetate concentrations have been shown to inhibit neutrophil NET formation, and the GPR43 knockout mice failed to mount a proper GC-dependent immune response. CD8 T cells functionally benefit from increased acetate concentrations. That being said, there are a limited number of investigations into specific effects of acetate on immunity. Many studies address acetate as a part of SCFA mixture, justified by the SCFA mixtures produced by gut microbiota. In our laboratory, we have shown a major effect of acetate on the immune system, which is discussed later in this review.

## 3. Alcohol and Acetate Affect Humoral Autoimmunity

### 3.1. Alcohol and Its Metabolite, Acetate, Reduce IL-21-Producing T_FH_ Cells

Alcohol’s effect on multiple autoimmune diseases have been well recognized. Alcohol consumption affects all aspects of immune system [9]. In particular, many RA human cohorts have demonstrated an inverse relationship between alcohol consumption and RA disease severity [23]. The protective effect of alcohol in RA was also proven to be true in a mouse model of RA, CIA [52,54]. Despite significant protection against disease, there are no specific studies on the mechanisms of alcohol’s action on immunity during inflammatory arthritis. In our laboratory, we aimed to delineate alcohol’s effects by investigating alcohol’s main metabolite, acetate, as well. Consumed alcohol is metabolized to acetate, and in turn, acetate is metabolized to acetyl-CoA [3,101]. In fed state, generation of additional acetyl-CoA leads to an expansion of secondary functions of acetyl-CoA [101]. It was therefore interesting to follow the effects of acetate on the immune responses during CIA. T_FH_ cells play a central role in supporting B cell activation, class switch, affinity maturation, and GC maintenance [102,103]. We have shown that, in the context of CIA, alcohol-fed mice exhibited no significant changes to CD4^+^ T cell populations aside from reduced Treg cells in the spleens and dLNs [54]. Increased T_FH_ cells have been shown to be increased in various autoimmune diseases [104,105,106]. The functionally active IL-21^+^ T_FH_ cell population was reduced in alcohol-consuming CIA mice. These findings were also confirmed by an in vitro naïve CD4^+^ T cell differentiation [54]. T_FH_ cells exhibited reduced IL-21 production upon alcohol and acetate treatment. IL-21 has been shown to drive RA disease in multifaceted manner, which is why there are multiple compounds in clinical trials targeting IL-21, such as tocilzumab and baricitinib [107]. In CIA, blocking of IL-21/IL-21R receptor pathway had significant protections against disease severity [108]. Increases in other molecules of T_FH_ cells, such as BCL-6, PD-1, CXCR5, and ICOS, have all been correlated with increased DAS28 scoring and ACPA antibody titers [105,109]. Upon alcohol consumption, as well as elevated acetate levels, all of these factors, relating T_FH_ cells to increased DAS28 scores, aside from ICOS and CXCR5, were reduced [54]. In addition, circulating T_FH_-like cells have been reported in the blood and synovium of RA patients [110,111,112]. It is plausible that reduced B cell activation, differentiation, GC formation, antibody class switch, and titers are the result of a reduction in IL-21^+^ T_FH_ cell function as IL-21 has been shown to be a major effector during GC reactions [113]. One major indicator of disease severity is existence of pathogenic antibodies [12,114,115]. It has been shown that serum ACPA and RF antibodies can be detected up to 20 years before the onset of RA and are used in diagnosis of RA [115,116]. Alcohol consumption has been reported to increase antibody titers in patients with alcoholic liver disease (ALD) in comparison to alcoholic patients without ALD [72]. However, reasons for increased antibody titers, whether being due to impaired antibody clearance or production, has not been revealed. Decreased antibody titers are also evidenced in hepatitis B vaccination of alcoholic patients, as they, too, had decreased IgG titers [117]. Alcohol-fed CIA mice have been shown to exhibit decreased CII-specific IgG and B cells [54]. In another study, the onset of CIA was significantly delayed along with decreased severity in mice treated with anti-mouse CD20 mAb targeting and depleting B cells [118,119]. Antibodies are the main factor augmenting and propagating the autoimmune response [115,120]. Even in mice without T and B cells, transferring CII-specific antibodies induced arthritis [121]. Acetate feeding of CIA mice has provided the same protective effect as observed with alcohol feedings [53,54].

### 3.2. The Effects of Alcohol/Acetate on T–B Cell Relationship

In a recent study of type 1 diabetes, for example, it was discovered that the newly diagnosed patients harbored less SCFA-producing microbiota and that the acetate in particular suppressed the generation of GC B cells [122]. In a type 1 diabetes mouse model, non-obese diabetic (NOD) mice fed with high acetate yield feed exhibited less autoreactive CD8^+^ and CD4^+^ T cells in comparison to NOD mice fed normal chow [123]. In the same study, a high acetate-yielding diet also reduced the frequency and numbers of splenic IgM^+^ B220^+^ B cells, as well as marginal zone B cells [123]. Although acetate alone has been shown to mitigate immune cells, high-fiber diet, which produces a mixture of SCFAs, has also increased T_FH_ cells and GC B cell numbers as well as increased IgA production [92]. It seems that acetate has immune-suppressive effects, while a mix of SCFAs including acetate have various effects, wherein the effects of one SCFA might overcome or undermine another. Because IL-21 is produced by T_FH_ and Th17 cells, it would be interesting to investigate IL-21 production by Th17 cells. In our study, CIA mice hydrodynamically injected with IL-21 mini circle DNA exhibited increased systemic levels of IL-21 and increased Th17 cell population. This increase, amongst other possible effects of IL-21, could explain the diminishing protective effect of alcohol. As there was no decrease in T_FH_ cell frequency in alcohol-fed CIA mice and due to the fact that T_FH_ cells increase IL-21 production upon interaction with B cells at the T:B zone border, we decided to analyze T_FH_:B cell conjugates [54,102,124]. We found that functionally active IL-21^+^ T_FH_:B cell conjugates were reduced upon alcohol exposure to CIA mice, as well as to IL-21-overexpressing CIA mice, indicating that alcohol’s effects manifest themselves upstream of T_FH_:B cell interaction. One plausible hypothesis was that alcohol-induced reduction in PD-1 affects T_FH_ cell positioning, as PD-1 was shown to be required for T_FH_ cell positioning in SLOs [125]. Previously, it was shown that alcohol significantly reduced the migration of DCs to LNs post-activation as Langerhans’ cells [60]. Such reduction of the DC population in SLOs was also observed in the case of alcohol-fed CIA mice in our study [54]. In addition to reduced DCs at the lymph nodes, it was previously found that antigen presentation by spleenocytes, DCs, and B cells was reduced upon alcohol exposure [56,75]. Interestingly, we found that upon alcohol or acetate feeding of CIA mice, there was a stark reduction in B cell follicle-infiltrating T cells. Moreover, in vitro T_FH_:B cell interaction and interaction stability was compromised upon alcohol and acetate exposure. Reduction in antigen presentation by B cells to T_FH_ cells could possibly contribute to reduced T_FH_:B conjugate formation [126]. Future investigations are needed to shed light on alcohol’s and acetate’s effects on cellular interactions.

## 4. Conclusions

**Figure 1:**
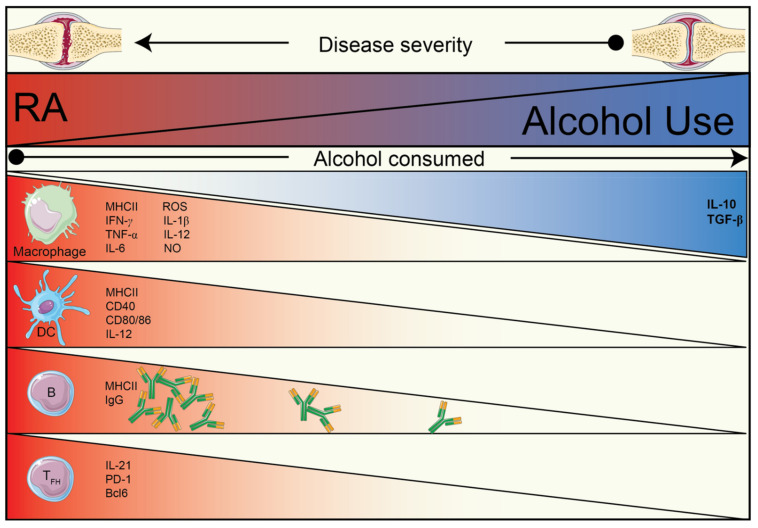
Summary of the inverse relationship of alcohol use and RA, grouped by known effects of alcohol and/or of its metabolites on different immune cells.

RA is a complex and multifactorial disease. Alcohol and its metabolites are potent modulators of many processes in various tissues and organs. It is undeniable that elucidating the mechanisms by which alcohol consumption protects against RA is a complicated task. In this review, as outlined in Figure 1, we attempted to bring all possible and proven facts of alcohol’s effect on the immune system within the scope of autoimmune arthritis. In short, the following factors have been found to be reduced upon alcohol exposure: antigen-presentation, T cell activation capacity of APCs, B cell maturation and proliferation, IL-21 production by T_FH_ cells, antigen-specific IgG, and proinflammatory cytokines; moreover, the following factors seem to be augmented: Th2 immune response, M2 macrophage function, and anti-inflammatory cytokines (IL-10, TGF-β). Effects mentioned here on different components of the immune system act in synergy, ultimately providing observable and measurable protection against RA.

## Figures and Tables

**Table 1:** Classification of alcohol consumption according to National Institute on Alcohol Abuse and Alcoholism (NIAAA).

	Moderate Consumption		Heavy Consumption	
	Men	Women	Men	Women
Alcohol in grams/day	<28	<14	>56 ^#^	>42 ^#^

^#^ on any day.
